# Programmable cell aggregation by a synthetic biosilicification approach

**DOI:** 10.1016/j.isci.2025.112519

**Published:** 2025-05-26

**Authors:** Qing Wang, Jing Sun, Lei Zang, Huaxiong Yao, Lin Wang, Runtao Zhu, Jie Li, Simin Zeng, Hongting Tang, Teng Wang, Ji Liu, Bo Wang, Bo Li, Zhiyuan Liu, Zhuojun Dai

**Affiliations:** 1 State Key Laboratory of Quantitative Synthetic Biology, Shenzhen Institute of Synthetic Biology, Shenzhen Institutes of Advanced Technology, Chinese Academy of Sciences, Shenzhen 518055, China; 2 School of Chemistry and Chemical Engineering, Harbin Institute of Technology, Harbin 150001, China; 3 Center of Neural Engineering, CAS Key Laboratory of Human-Machine Intelligence-Synergy Systems, Shenzhen Institute of Artificial Intelligence and Robotics for Society, Shenzhen Institutes of Advanced Technology, Chinese Academy of Sciences, Shenzhen 518055, China; 4 Department of Mechanical and Energy Engineering, Southern University of Science and Technology, Shenzhen 518055, China; 5 Department of Civil Engineering, University of Nottingham Ningbo China, 199 Taikang East Road, Ningbo 315100, China

**Keywords:** Bioengineering, Synthetic biology, Biomaterials

## Abstract

Programmable cell aggregation offers valuable insights into the natural development of synthetic multicellular systems and enables precise control over spatial organization and material structuring. Previous efforts have focused on modifying cells with designed organic-based adhesive modules and assembling cells into defined patterns. Here, we present a different approach to guide cell assembly by tuning the organic-inorganic interactions. Our method involves engineering cells to express a silicifying peptide on their surfaces, which promotes silica deposition on the cell walls. This peptide simultaneously binds to the silica synthesized on adjacent cells, triggering cell clustering. The engineered cells exhibit rapid aggregation, with approximately 95% of cells assembling within 15 min. We further show that this capability can facilitate materials assembly and chemical production. Our biosilicification-based approach offers novel insights into natural multicellularity mechanisms and holds potential for applications in biomanufacturing and materials engineering.

## Highlights

• Programmable cell assembly by engineered organic-inorganic interactions
• Development of a mathematical model to predict aggregation kinetics
• Assembly of living building materials by mixing sand with engineered bacteria
• Manufacturing of chemicals with oxygen-sensitive pathway

## Graphical abstract

**Figure:**
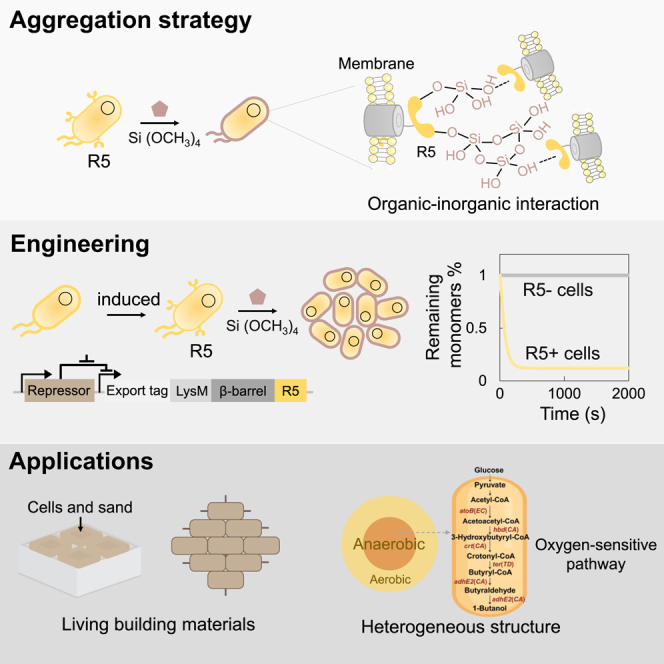

## Introduction

Cell-cell aggregation is widely known as an essential process of cell-cell interactions to form multicellularity.^1^^,^^2^ These multicellular systems often display a variety of morphologies (three-dimensional structures) across multiple length scales with associated biofunctions. Therefore, controlling cell-cell aggregation can enhance our understanding and engineering of various cellular processes, such as spatial organization and the creation of structured living materials. By far, numerous strategies have been developed to achieve programmed cell-cell aggregations. Most methods employ chemical reagents, including compounds, polymers, and biomacromolecules (proteins and nucleic acids), to modify cell surfaces and act as “cell glue” for adhesion.^3^^,^^4^^,^^5^^,^^6^^,^^7^ However, these non-genetic methods require chemical modifications and are limited by the continuous dilution of functional moieties due to cell growth. In contrast, some genetic approaches to direct cell-cell adhesion have been developed, facilitating aggregation through interactions of specific proteins expressed on the cell surface.^1^^,^^2^^,^^8^^,^^9^ Currently, most adhesive modules are bio-organic based, and the tools to direct aggregation remain underdeveloped.

In nature, numerous delicate structures arise from organic-inorganic interactions. For example, silica-condensing microorganisms such as diatoms mineralize their tissues to form complex, functional architectures at various scales.^10^^,^^11^ Interestingly, Xiong et al. reported an engineered cell aggregation method by mimicking biological silicification. They coated the cells with poly(diallyldimethylammonium chloride), which simulated silicification proteins to induce *in situ* silica deposition onto cell surfaces.^12^ This resulted in unspecific self-aggregation of the silicified *Chlorella pyrenoidosa* cells due to the surface charge neutralization. This novel approach introduced inorganic-based biomineralization to program cell aggregation, albeit it was non-genetic and required additional macromolecules and cell modification.

**Figure 1:**
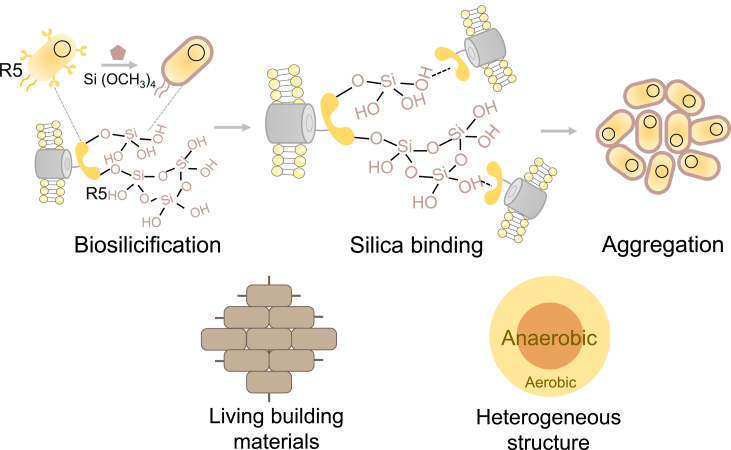
Engineered silicification enables programmable aggregation *E*. *coli* cells are engineered to display a synthetic silaffin peptide facilitating silicification under gentle condition. The cell surface-bound R5 peptide on one cell can simultaneously adhere to the synthesized silica on neighboring cells, inducing their aggregation. This dual functionality fosters the creation of living building materials and advances biomanufacturing capabilities. In biomanufacturing, cells in the exterior layer of the aggregate act as a shell to prevent the penetration of ambient O_2_ into the core, creating a hypoxic domain for the cells in the inner layer and facilitating the biomanufacturing of chemicals with the pathway sensitive to the oxygen.

By leveraging the tools from synthetic biology, here we developed a genetic-based biosilicification method to direct cell aggregation. We engineered *Escherichia coli* to display a synthetic silaffin peptide, R5 (SSKKSGSYSGSKGSKRRIL), due to its dual functionalities in mediating silicification and its binding affinity toward silica.^13^^,^^14^^,^^15^^,^^16^ The peptide enabled cell mineralization and facilitated silica synthesis on the cell surface, while the surface-bound peptide tethered to the silica precipitated on neighboring cells, driving cell clustering (Figure 1). Based on this design, we further constructed living building materials and created structured multicellular complex for versatile biomanufacturing.

## Results

### Programmable aggregation induced by biosilicification

**Figure 2:**
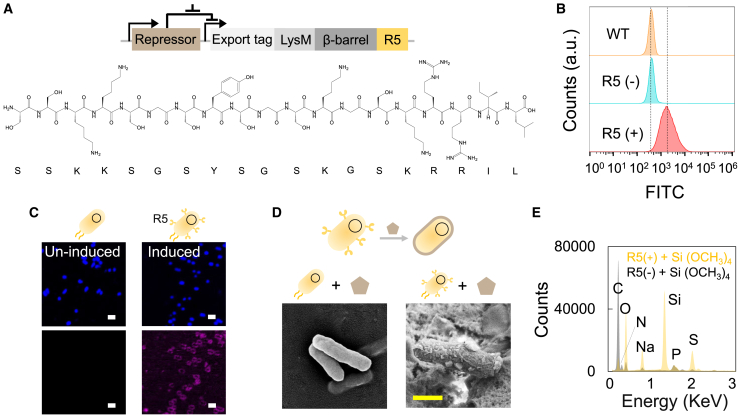
Biosilicification mediated by R5 peptide display (A) Circuit architecture and amino acid sequence of the R5 peptide. The circuit design incorporates the R5 peptide sequence regulated by a TetR repressor, which is controlled by anhydrotetracycline (aTc) induction. (B) Cell engineering for R5 peptide display regulated by an inducible gene circuit. Flow cytometry analysis demonstrated effective R5 peptide display on cells upon induction. Panels display wild-type cells (top), uninduced cells (middle), and induced cells (bottom). (C) Immunostaining analysis of bacteria suggested the presence of R5 on the cell surface. The left and right columns represent uninduced and induced cells, respectively. The first row shows Hoechst 33342 staining (a universal DNA dye), while the second row shows immunostaining targeting the His-tagged R5. Scale bars, 2 μm. (D) Biosilicification of engineered cells. Induction of biosilica deposition was achieved by incubating R5-displaying cells with Si(OCH_3_)_4_, resulting in silica deposition on the cell surface. Left and right images show surface morphology of uninduced or induced bacteria after incubation with Si(OCH_3_)_4_, respectively. Scale bars, 1 μm. (E) Energy-dispersive spectroscopy (EDS) analysis confirmed silicon presence on the cell surface of induced cells. In contrast, no silicon was detected on uninduced cells incubated with Si(OCH_3_)_4_.

We firstly designed a biosilicification circuit in *E*. *coli* (Figure 2A). This circuit contained a transcriptional regulator, an outer membrane anchor, and a synthetic silaffin peptide.^1^ The regulator utilized a standard TetR repressor, controlled by anhydrotetracycline. The outer membrane anchor was constructed by an intimin display system containing a short N-terminal signal peptide (export tag) for trafficking to the periplasm, a LysM domain for peptidoglycan binding, and a β-barrel for transmembrane insertion.^17^^,^^18^^,^^19^ We fused a 19-mer R5 peptide, derived from silaffin peptides in the diatom *Cylindrotheca fusiformis*, on the C terminus of the truncated intimin^13^^,^^20^ (Figure 2A). After transforming the circuit into *E*. *coli*, we verified R5 peptide display through immunostaining, by incubating the cells (induced [R5+], uninduced [R5−], and wild-type cells [WT, not carrying the circuit]) sequentially with primary antibodies against the fused His-tag and fluorophore-conjugated secondary antibodies (Figures 2B, 2C, S1, and S2). Both the flow cytometry and confocal microscopy results showed that the fluorescence from immunostaining appeared on the cell wall exclusively for cells with induction, indicating the successful surface tethering of the R5 peptide (Figure 2C).

**Figure 3:**
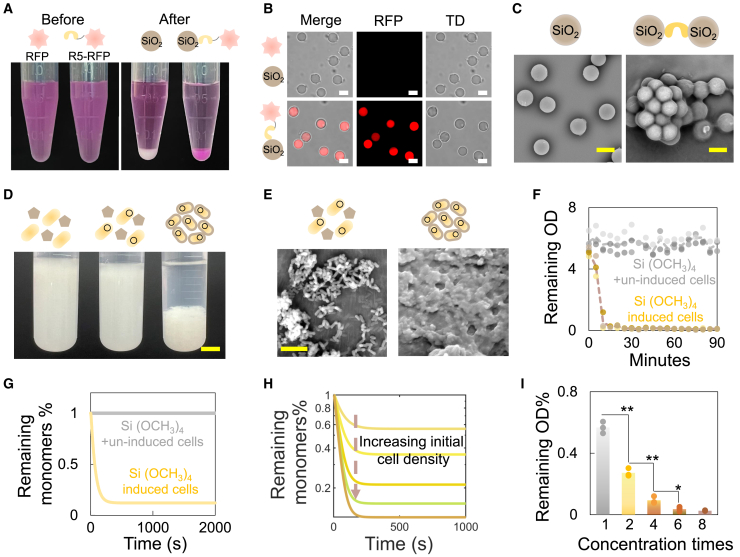
Programmable aggregation induced by the dual functionality of R5 peptide (A) R5-fused protein affinity for silica. Purified RFP and R5-fused RFP (left image) were tested for binding affinity to silica microspheres (right image). After incubation, microspheres incubated with R5-fused RFP turned red (right tube), confirming R5’s affinity for silica. (B) Microscopy verification of R5-fused RFP conjugation to silica. Columns represent merged, RFP and transmitted light channels, respectively. Scale bars, 5 μm. (C) R5-induced aggregation of silica microspheres. Images taken by scanning electron microscope (SEM) show microspheres in deionized water (left) and microspheres with added purified R5 (right), indicating R5-driven clustering. Scale bars, 5 μm. (D) Programmable cell aggregation via biosilicification. *E*. *coli* cells (wide-type [left], uninduced [middle] and induced [right]) were resuspended in PBS (OD_600_ ∼ 5.5) and incubated with Si(OCH_3_)_4_. Pictures were taken at 30 min. Scale bars, 1 cm. (E) SEM analysis of dispersed and aggregated cells. SEM images depict uninduced (left) and induced cells (right) after reaction with Si(OCH_3_)_4_, with induced cells showing aggregation. Scale bars, 4 μm. (F) Cell settlement dynamics tracking. OD_600_ in the supernatant was measured every 5 min to monitor the aggregation dynamics over time. Dashed line = mean value (*n* = 3). (G) Aggregation dynamics simulation via mathematical modeling. A kinetic model simulating silicified cell aggregation was developed (see STAR Methods), assuming cells become aggregation-prone post silicification. (H) Impact of initial cell density on aggregation dynamics predicted by the simulation model. The simulation model indicates that higher initial cell density promotes more efficient aggregation. Initial cell densities were set at 0.002, 0.004, 0.008, 0.012, and 0.016 (from top to bottom) for the plotted curves. (I) Tuning aggregation dynamics through initial cell density. Aggregation efficiency was assessed by the percentage of remaining OD_600_ in the supernatant after 1.5 h, normalized to initial OD_600_ at time zero. The initial OD_600_ of 1× culture is ∼2.8. ∗∗ or ∗ indicates *p* < 0.01 or 0.05 (*p* = 0.0007, 0.0010, and 0.0306 for 2× culture compared with 1× culture, 4× culture compared with 2× culture, 6× culture compared with 4× culture, as determined by the two-sided Student’s two-sample t test assuming unequal variances. Column = mean value [*n* = 3]).

Although the development of intricate silica structures of diatoms is still regarded as a paradigm, it is generally believed that silicic acid in surface water or oceans is imported via specific transporter proteins and then binds with polypeptide silaffins to form silica aggregates.^21^ To mimic this process, we supplemented tetramethyl orthosilicate (TMOS, Si(OCH_3_)_4_) directly to the R5-displayed cells. The nucleated silica particles were observed and confirmed through analysis by scanning electron microscopy and energy-dispersive spectroscopy, validating the capability of R5 to mineralize silica on non-native species (Figures 2D, 2E, and S3). Another key trait of R5 peptide is its binding affinity toward silica. To verify this, we expressed the R5-tagged RFP in *E*. *coli* and incubated the purified proteins with silica microspheres. The conjugation between R5 and silica resulted in a visually observable color change of microspheres. In comparison, no color change occurred when incubating the substrate with purified RFP lacking R5 (Figures 3A, 3B, and S4). The dual functionalities of R5 suggest its potential in mediating cell-cell interactions through organic-inorganic binding, as anchored R5 can both deposit silica and adhere to silica on neighboring cells. Supplementing purified R5 peptides to silica microspheres indeed led to system aggregation, partially justifying our assumption (Figures 3C, S5, and S6).

We then applied the system to living cells. Stationary-phase cultures, including WT cells and engineered cells (carrying the R5 display circuit) with or without induction, were left unshaken. Upon the addition of TMOS, cell-cell interactions became evident through visible aggregation and settling (Figures 3D and 3E). In contrast, no aggregation was observed in either WT or uninduced cells, even with TMOS added (Figures 3E and S7). To track aggregation dynamics, we measured the optical density at 600 nm (OD_600_) of cells in the upper part of the cultures (the non-aggregated fraction). Results indicated that approximately 95% of cells settled within 15 min (Figure 3F). The kinetics of aggregation could be captured by an ordinary differential equation-based mathematic model (Figure 3G). Specifically, the model predicted that the efficacy of the aggregation could be modulated by tuning the initial cell number: higher initial cell densities would accelerate the aggregation process (Figure 3H). To verify this prediction, we added a fixed amount of TMOS to cultures with varying initial cell densities and monitored the settling process. As anticipated, the percentage of cells remaining in the non-aggregated fraction after one and half hour decreased with higher initial cell densities, demonstrating the tunability of the aggregation process (Figure 3I).

### Living materials assembly

**Figure 4:**
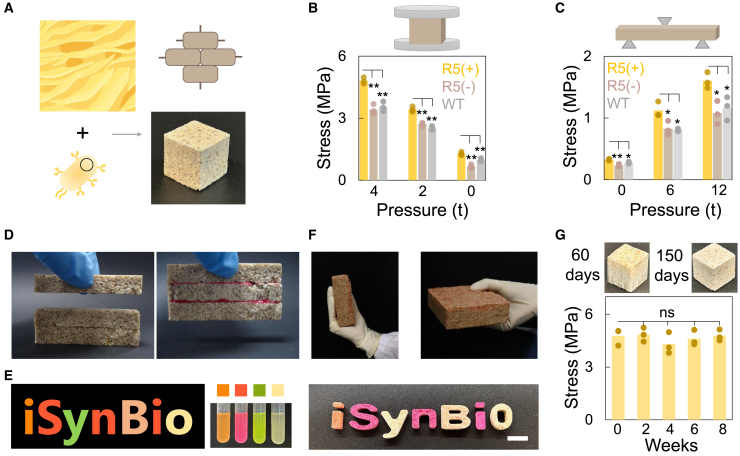
Fabrication of living building materials (A) Bio-bricks using engineered cells and sand. Bio-bricks were created by mixing engineered cells displaying the R5 peptide with sand. The resulting cubic blocks measured 1 cm × 1 cm × 1 cm. (B) Compressive strength of bio-bricks. Bio-bricks made with induced R5-displaying cells and sand showed enhanced compressive strength compared to those made with uninduced or wild-type cells (∗∗ indicates *p* < 0.01 [*p* = 0.0021, 0.0025, and 0.0009 for R5(+) compared with R5(−) when pressure equals 4, 2, or 0 ton; *p* = 0.0029, 0.0010, and 0.0086 for R5(+) compared with WT when pressure equals 4, 2, or 0 ton, as determined by the two-sided Student’s two-sample t test assuming unequal variances; column = mean value (*n* = 3)]). Increasing preparation pressure further improved mechanical properties. The cell-to-sand ratio was 3.6:20 (w/w). (C) Flexural strength evaluation. Bio-bricks for flexural testing had a cuboid shape (6 cm × 1 cm × 1 cm) with a cell-to-sand ratio of 3.6:20 (w/w). ∗∗ or ∗ indicates *p* < 0.01 or 0.05 (*p* = 0.0050, 0.0413, and 0.0210 for R5(+) compared with R5(−) when pressure equals 0, 6, or 12 tons; *p* = 0.0152, 0.0468, and 0.0366 for R5(+) compared with WT when pressure equals 0, 6, or 12 tons, as determined by the two-sided Student’s two-sample t test assuming unequal variances. Column = mean value [*n* = 3]). (D) R5-displayed cells as adhesive for bio-bricks assembly. Each bio-brick weighed approximately 10 g (6 cm × 1 cm × 1 cm). To join three bio-bricks vertically, ∼2 g of R5/mScarlet-expressing cells were applied to their contact surfaces. After 1 day, the adhesive properties of the R5/mScarlet-expressing cells formed a stable combined brick (∼30 g), which could be lifted entirely by applying force to the top unit alone. (E) Programmable color in bio-bricks. Bio-bricks were programmed with cells expressing various fluorescent proteins (tubes from left to right containing cells carrying R5/mOrange, R5/mScarlet, R5/GFP, or R5, respectively) to create colored letters. The letter “i” was made with R5/mOrange and R5/mScarlet cells (1:1 ratio). Letters “S,” “n,” and the right “i” used R5/mScarlet cells. “y” was made with R5/GFP cells, “B” with R5/mOrange cells, and “o” with R5 cells. Scale bars, 2 cm. (F) Large bio-bricks fabrication with engineered cells. A larger bio-brick (10 cm × 10 cm × 3 cm) was produced by mixing approximately 21.6 g of R5-displaying cells (R5/mScarlet and R5/mOrange cells in a 1:1 ratio) with 600 g of sand. (G) Stability of bio-bricks over time. Bio-bricks retained stable compressive strength (ns indicates not significant comparing the compressive strength of bio-bricks at week 0 and other times) and shape over time. Week 0 was set as the third day after fabrication to allow for adequate moisture evaporation. The cubic blocks measured 1 cm × 1 cm × 1 cm.

In nature, multiple biological systems can generate structured materials that integrate organic and inorganic elements, yielding diverse physical and chemical properties. The capability of R5 to mediate organic-inorganic interactions offers valuable opportunities for materials assembly. The system is especially advantageous for constructing building materials due to R5’s binding affinity toward silica, which is abundant as sand in nature (Figure 4A). Sand is one of the most widely used natural resources and fundamental ingredient in mortar, concrete, and other building composites. However, the negative environmental impact of traditional building materials like cement, responsible for 8% of global greenhouse gas emissions, highlights the pressing need for sustainable alternatives.^22^ While previous studies have explored hydrogels as binders for load-bearing structures, these solutions require additional synthetic processes and pose challenges in sustainability.^23^

We utilized genetically engineered cells as organic binders to create sand-based structural materials. Experimental results confirmed that R5-tagged proteins could adhere to sand, as evidenced by the enhanced fluorescence under microscopy (Figures S8 and S9). We then screened various engineered bacteria-to-sand ratios, analogous to binder-to-aggregate ratios in traditional concrete, and evaluated the mechanical properties through compressive strength measurement. The engineered cells and sand were mixed and molded into cube-shaped structures (Videos S1 and S2). Within 2 days, most excess moisture had evaporated from the cubes (Figure S10). Results suggested that a higher binder concentration contributed to the increased mechanical property of the sample (Figure S11), underscoring the binding capability of the engineered cells. We generated both the strength-strain curves (compressive test) and flexural strength-strain curves (three-point bending test) of the samples made by blending the sand with various cells (induced [R5+], uninduced [R5-], or WT cells [not carrying the circuit]) (Figure S12). Cubes generated by sand blended with R5-displayed cells demonstrated superior compressive and flexural properties in both tests (Figures 4B and 4C). Additionally, the presence of R5-displaying bacteria enabled adhesion between bio-bricks, providing a self-healing capability for repairing cracks in building materials (Figure 4D).

Video S1. Sand could not be molded into a specific shape

Video S2. The mixture of sand and R5-displayed cells was molded into a cubic

We leveraged cell programmability to fine-tune the living materials further. Specifically, we engineered R5-displaying cells to co-express various fluorescent proteins and then combined these cells with sand to produce visibly colored materials (Figure 4E). The object size was adjustable; for instance, we mixed approximately 21.6 g of cells with 600 g of sand to fabricate a bio-brick measuring 10 cm × 10 cm × 3 cm (Figure 4F). The materials were relatively stable. We further tracked the compressive strength of the bio-brick in 8 weeks and observed only limited changes. The structure maintained its integrity even after 150 days, certifying the robustness of the system (Figure 4G).

### Programmable aggregation for biomanufacturing

**Figure 5:**
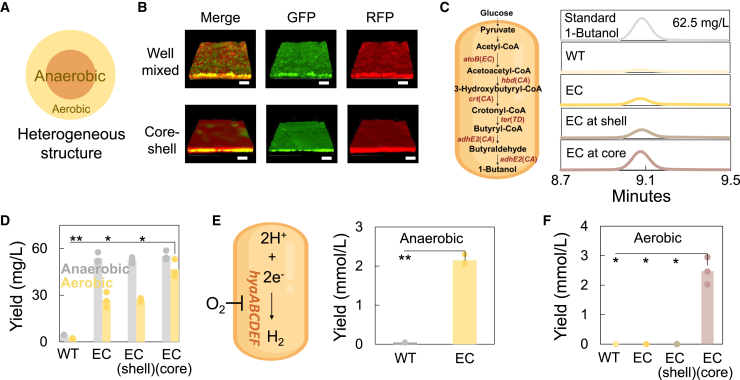
Enhanced biomanufacturing of chemicals via heterogeneous structure (A) Formation of structured aggregation through the programmable biosilicification. Cells in the outer shell consumed oxygen, creating an anaerobic core for the cells in the inner layer. (B) Generating the structured aggregation by sequentially layering the cells. Cells expressing R5/GFP (core) were first sedimented, followed by the addition and settlement of R5/RFP cells (shell) to create the structured aggregation (bottom row). In contrast, simultaneous settling of both cell types resulted in uniformly mixed fluorescence (top row). Scale bars, 10 μm. (C) 1-butanol production by structured aggregation. A 1-butanol synthesis pathway was engineered in *E*. *coli* BL21(DE3) (left). Gas chromatography (GC) confirmed 1-butanol production (right), with panels showing, from top to bottom, a standard (62.5 mg/L), wild-type cells (no pathway), engineered cells (ECs, cells carrying the pathway), ECs in the shell layer (ECs at shell, cells carrying the pathway in the shell layer of the aggregate), and ECs in the core layer (ECs at core, cells carrying the pathway in the core layer of the aggregate). (D) Quantification of 1-butanol yield in aerobic or anaerobic environments. ECs in the core layer of the aggregate (EC(core)) achieved a significantly higher 1-butanol yield in aerobic environments compared to wild-type cells, non-structured ECs, and ECs in the shell layer of the aggregate (EC(shell)). ∗∗ and ∗ indicate *p* < 0.01 or 0.05 (*p* = 0.0054, 0.0106, and 0.0229 for EC(core) compared with WT, ECs, and EC(shell), as determined by the two-sided Student’s two-sample t test assuming unequal variances; column = mean value [*n* = 3]). (E) Biomanufacturing of hydrogen by ECs. An engineered hydrogen production pathway in *E*. *coli* BL21(DE3) enabled hydrogen generation under anaerobic conditions. Column = mean value (*n* = 3). ∗∗ indicates *p* < 0.01 (*p* = 0.0013 for ECs compared with wild-type cells, as determined by the two-sided Student’s two-sample t test assuming unequal variances; column = mean value [*n* = 3]). (F) ECs in the core layer of the structured aggregate were capable of producing hydrogen even in aerobic environment. No hydrogen was detected for wild-type cells, non-structured ECs, or ECs in the shell layer of the aggregate (EC(shell)), due to the oxygen sensitivity of hydrogenase. ∗ indicates *p* < 0.05 (*p* = 0.0114, 0.0114, and 0.0114 for EC(core) compared with WT, ECs, and EC(shell), as determined by the two-sided Student’s two-sample t test assuming unequal variances; column = mean value [*n* = 3]).

The self-aggregation of engineered bacteria can facilitate the collection of cell mass during fermentation. At the same time, the aggregate can be considered to be an autogenously structured complex with differentiated cell functions. Cells in the exterior layer (shell part) act as a shell to prevent the penetration of ambient O_2_ into the core and further consume the diffused O_2_ to create a hypoxic domain for the cells in the inner layer (core part), facilitating the biomanufacturing of chemicals with the pathway sensitive to the oxygen (Figure 5A).^12^^,^^24^

We first explored a reliable protocol to assemble a differentiated structure utilizing silicified cells. Simply mixing two types of cells expressing different fluorescent proteins produced a homogeneous fluorescence distribution. However, by sequentially layering two different silicified cells, we achieved distinct separation of fluorescence in the core (green) and shell (red) regions, indicating autonomous formation of the desired structure (Figures 5B and S13). We continued to rewire the cells to produce specific value-added chemicals. Especially, we chose two different chemicals, 1-butanol and hydrogen, due to their oxygen-sensitive pathways. 1-Butanol is an important chemical feedstock used in surface coating, plastics, and fuel substitutes. We constructed the synthetic pathway in *E*. *coli* for efficient production of 1-butanol (Figure 5C). The pathway harnessed acetyl-CoA acetyltransferase (*Ec*-atoB) from *E*. *coli*, 3-hydroxybutyryl-CoA dehydrogenase (*Ca*-hbd) from *Clostridium acetobutylicum*, short-chain-enoyl-CoA hydratase (*Ca*-crt) from *C*. *acetobutylicum*, the electron donor (*Td*-ter) from *Treponema denticola*, and aldehyde-alcohol dehydrogenase (*Ca*-adhE2) from *C*. *acetobutylicum*, among which the aldehyde-alcohol dehydrogenase was particularly sensitive to oxygen and turned partially inactive with the existence of oxygen.^25^^,^^26^ Culturing the engineered cells in the aerobic condition, instead of the anaerobic condition, resulted in limited production of 1-butanol (Figures 5C and 5D). We then generated the aggregation with spatial-functional differentiation by sequentially layering the cells carrying both the pathway and R5 display circuit (core region) and cells carrying only the R5 display circuit (shell region). Even when cultured aerobically, cells in the shell layer of the aggregate gradually consumed oxygen through aerobic respiration, producing an anaerobic environment for the *E*. *coli* cells carrying the pathway in the core. Consequently, the system attained a higher yield with ∼46.7 mg/L 1-butanol, compared with the cases of engineered cells without structuring (∼26.8 mg/L) or reversing the cells in the core and shell region (∼27 mg/L), as quantified by the gas chromatography measurement (Figures 5D and S14A).

Several research studies have developed biological systems for hydrogen production. However, the oxygen-sensitive nature of these reactions and the low yield of the isolated hydrogenase have posed challenges, highlighting the need for further system improvements.^24^^,^^27^ To overcome the low hydrogenase yield, we overexpressed the hydrogenase in engineered *E*. *coli* (Figure 5E, left). Under anaerobic conditions, the rewired cells produced substantially more hydrogen compared to WT cells (Figure 5E, right). In contrast, neither system produced hydrogen under aerobic conditions due to the oxygen sensitivity of hydrogenase. We further generated the structured aggregation with spatial-functional differentiation by sequentially layering the cells expressing both the hydrogen pathway and R5 display circuit (core region) and cells carrying only the R5 display circuit (shell region). Within the structured aggregation, engineered cells showed an increased hydrogen production and reached ∼2.47 mM at 24 h when cultured aerobically, underscoring the protection ability of the structure to maintain the system’s catalytic activity (Figures 5F, S14B, and S15). In comparison, both the non-structured engineered cells and the structured aggregation reversing two cells in the core and shell regions produced no hydrogen due to exposure to oxygen (Figures 5F and S14B).

## Discussion

In this work, we established a programmable, genetic-based cell aggregation strategy based on organic-inorganic interactions. This approach leverages the dual functionality of the R5 peptide, which not only facilitates silica deposition but also enhances the binding affinity of silicified cells. The aggregation process is highly efficient, with approximately 95% of cells settling within the first 15 min (initial OD_600_ ∼ 5.5). The efficacy of cell aggregation is tunable by varying the initial cell concentration. This method, based on the mild peptide-silica interactions, can be compatible with various cell strains or species, broadening the applicability of the system.

To explore the applications of the system, we used the engineered cells to assemble living building materials and construct the structured aggregation for biomanufacturing. Recently, a novel cement-free living building material was developed. It relied on a bacteria-inoculated “scaffold” made of desiccated gelatin hydrogel to bind sand aggregates.^23^ Bacterial cells further toughened the system via microbial-induced calcium carbonate precipitation. Comparatively, our method does not require an external scaffold. Instead, it utilizes fast-growing, self-replicating engineered cells as a natural binder to adhere sand particles together. The system is stable, with the structure maintained for over 5 months. The approach is flexible, allowing for the use of cells capable of precipitating calcium carbonate or other minerals to further enhance the mechanical properties.

The structured aggregation protects the activity of oxygen-intolerant enzymes in the core region, enabling efficient biological whole-cell catalysis under simple, mild conditions in ambient air. The method is adaptable and can incorporate various metabolic pathways, making it suitable for diverse biomanufacturing applications. Together, we envision that the system could inspire or benefit multiple areas ranging from materials assembly, pattern formation, and chemical production.

### Limitations of the study

Current living building materials are primarily composed of engineered cells and R5. However, the system lacks waterproofing, causing it to lose mechanical strength and structural integrity when submerged in water. Future research could explore the incorporation of water-resistant coating materials or further genetic engineering of cells to produce stronger adhesive compounds.

## Resource availability

### Lead contact

Further information and requests for resources and materials should be directed to and will be fulfilled by the lead contact, Zhuojun Dai (zj.dai@siat.ac.cn).

### Materials availability

Requests for reagents will be fulfilled by contacting the lead contact upon reasonable request.

### Data and code availability

• Source data have been deposited at Mendeley Data and are publicly available as of the date of publication. DOIs are listed in the key resources table. All other data reported in this paper will be shared by the lead contact upon request.
• The source data and source code were deposited in Mendeley Data.
• Any additional information required to reanalyze the data reported in this paper is available from the lead contact upon request.

## Acknowledgments

This study was partially supported by the 10.13039/501100012166National Key Research and Development Program of China (2024YFA0920100) (Z.D.), the 10.13039/501100001809National Natural Science Foundation of China (32222047) (Z.D.), the Guangdong Natural Science Funds for Distinguished Young Scholar (2022B1515020077) (Z.D.), Shenzhen Medical Research Fund (grant no. D2401010) (Z.D.), the Strategic Priority Research Program of the Chinese Academy of Sciences (no. XDB0480000) (Z.D.), and the Shenzhen Science and Technology Program (ZDSYS20220606100606013 and KQTD20180413181837372) (Z.D.). We are grateful to the Shenzhen Infrastructure for Synthetic Biology for providing instrument support and technical assistance.

## Author contributions

Q.W., J.S., L.Z., and H.Y. designed and performed experiments, interpreted the results, and assisted in the manuscript writing. L.W. and R.Z. assisted in experimental setup, performing experiments, data interpretation, and manuscript writing. J. Li and S.Z. assisted in performing experiments and data analysis. H.T. assisted in experimental setup. T.W. assisted in mathematical model revision. J. Liu, B.W., and Z.L. assisted in experimental setup. Z.D. conceived the research, generated the mathematic model, assisted in research design and data interpretation, and wrote the manuscript.

## Declaration of interests

The authors declare no competing interests.

## STAR★Methods

### Key resources table

**Table:** 

REAGENT or RESOURCE	SOURCE	IDENTIFIER
**Bacterial strains**		

BL21(DE3) Chemically Competent Cell	Transgen	Cat#CD601-02
*Trans*1-T1 Phage Resistant Chemically	Transgen	Cat#CD501-02
*Escherichia coli* K12 MG1655	This study	N/A

**Chemicals, peptides, and recombinant proteins**		

Imidazole	Sigma-Aldrich	Cat#I5513-100G
Tween 20	Sangon Biotech	Cat#A600560-0500
Yeast extract	Sigma-Aldrich	Cat#V900886-500G
Agar	Sangon Biotech	Cat#A505255-0250
Glycerol	Sangon Biotech	Cat#A600232-0500
Isopropyl beta-D-thiogalactoside	Aladdin	Cat#I104812-5g
Ampicillin sodium	Aladdin	Cat#A105483-5g
Kanamycin	Solarbio	Cat#K8020-10g
Chloramphenicol	Aladdin	Cat#C100333-500g
Agarose	Biowest	Cat#BY-R0100-100g
Hydrogen chloride	Donguan dongjiang	N/A
Tert-Butanol	Macklin	Cat#T819476-500ml
Glutaraldehyde	Macklin	Cat#G916054-500ml
Sodium chloride	Sigma-Aldrich	Cat#S3014-500G
Trizma® base	Sigma-Aldrich	Cat#T1503-500G
Ethyl alcohol absolute	Yonghua Chemical	Cat#E101501-s5L
Phosphate-buffered saline (PBS)	Solarbio	Cat#P1000
LB Agar (Without Sugar)	Huankai Biology	Cat#028334
Tryptone	Macklin	Cat#T6276-500G
Dipotassium hydrogen phosphate	Sigma-Aldrich	Cat#P3786-500G
Potassium phosphate monobasic	Sigma-Aldrich	Cat#P0662-500G
Arabinose	Sangon Biotech	Cat#A422844-0005
D-Glucose	Sangon Biotech	Cat#A501991-0500
Ammonium phosphate dibasic	Sigma-Aldrich	Cat#A5764-500G
Potassium sulfate	Sigma-Aldrich	Cat#223492-500G
Magnesium sulfate heptahydrate	Sigma-Aldrich	Cat#230391-500G
Iron(II) sulfate heptahydrate	Sigma-Aldrich	Cat#215422-250G
Zinc sulfate heptahydrate	Sigma-Aldrich	Cat#221376-500G
Copper(II) sulfate pentahydrate	Sigma-Aldrich	Cat#209198-100G
Manganese sulfate monohydrate	Sigma-Aldrich	Cat#221287-100G
Calcium chloride dihydrate	Sigma-Aldrich	Cat#223506-500G
Sodium tetraborate decahydrate	Sigma-Aldrich	Cat#S9640-25G
Sodium selenite pentahydrate	Aladdin	Cat#S337199-25g
Ammonium heptamolybdate	Sigma-Aldrich	Cat#431346-50G
Ammonium nickel sulfate hexahydrate	Sigma-Aldrich	Cat#A1827-100G
1-Butanol	Sigma-Aldrich	Cat#360465-500ML
Ethyl acetate	Sigma-Aldrich	Cat#1133539030
Hydrogen	Hongzhou	N/A
LB Broth (Without Sugar)	Huankai Biology	Cat#028324
Hoechst 33342	Solarbio	Cat#C0031
Anhydrotetracycline hydrochloride	Macklin	Cat#A914394-100mg
Anti-6X His tag® antibody [HIS.H8]	Abcam	Cat#ab18184
Cy3-conjugated Goat anti-mouse IgG	Sangon Biotech	Cat#D110088-0100
FITC Anti-6X His tag® antibody	Abcam	Cat#ab1206
2X TransStart® FastPfu PCR SuperMix	Transgen	Cat#AS221-01
2X Basic Assembly Mix	Transgen	Cat#CU201
GelStain Blue (10000 X)	Transgen	Cat#GS102-01
50X TAE Buffer	Sangon Biotech	Cat#B548101-0500
Tetramethoxysilane(TMOS)	Aladdin	Cat#T110592-500g
Silica microspheres (5 μm and 200 nm)	Meryer	Cat#M85550-5ML
Chinese ISO standard sand	Xiamen Aisio	N/A

**Critical commercial assays**		

TIANprep Mini Plasmid Kit	Tiangen	Cat#DP103-03
E.Z.N.A.® Gel Extraction Kit	Omega	Cat#D2500-02
Ni Sepharose^TM^ 6 Fast Flow	Cytiva	Cat#17531801-25mL

**Oligonucleotides**		

Oligos for DNA assembly (Table S3)	This study	N/A

**Deposited data**		

Source data	This study	https://doi.org/10.17632/ywshy2r6dv.1
Software and algorithms	This study	https://doi.org/10.17632/ywshy2r6dv.1
Raw images	This study	https://doi.org/10.17632/ywshy2r6dv.2
Protein sequences	This study	https://doi.org/10.17632/ywshy2r6dv.1
Fiji	https://imagej.net/imagej-wiki-static/Fiji	v2.9.0/1.53t
NIS Elements AR	Nikon	v5.41.01
FlowJo	https://flowjo.bectondickinson.cn	v10.10

**Other**		

Confocal Dish	Solarbio	Cat#YA0572
Poly-L-Lysine-Prep Slides	Sangon Biotech	Cat#E678002-0000
Cover Glass	Sangon Biotech	Cat#F518114-0001
Carbon Double-Sided Tape	NISSIHIN EMCo.	Cat#1260729

### Experimental model and subject details

#### Bacterial strains, circuit and media

• All experimental data in main figures and supplementary figures were generated using *E*. *coli* strain BL21 (DE3). Plasmids were constructed in the *E*. *coli* strain Trans1-T1. The hydrogen production gene cluster was cloned from the genome of the *E*. *coli* K12 MG1655 strain.
• R5 peptide was first synthesized by Azenta, and then assembled into a display circuit published previously^1^. Briefly, R5 was anchored via a Neae surface display system which included a short N-terminal export tag, a LysM domain for peptide glycan binding, and a β-barrel for transmembrane insertion. Protein expression was controlled by a TetR operator (p15A origin).
• The sequence of *Ca-hbd*, *Ca-crt*, *Td-ter*, and *Ca-adhE2* were synthesized by Sangon Biotech with codon optimization.^28^ The fragments of *Ec-atoB* were amplified from *E*. *coli* BL21(DE3) genome. All gene fragments and the vector (araBAD promoter and p15A origin) were amplified and the final plasmid was constructed by Gibson assembly.
• *hyaABCDEF* gene was first amplified from the genome of *E*. *coli* MG1655, and then assembled into the vector with the T7 promoter and p15A origin.
• Relevant protein sequences can be found in Table S2. Primers used to construct the circuit can be found in Table S3.
• The experiments (when replicates were shown) were performed with 3 biologically independent samples. The sample sizes were chosen based on the scale of the project and consistency with other similar published studies. No sample size calculation was performed. Our results suggested that the sample sizes we chose were sufficient to test the theoretical predictions.

#### Growth media

**Luria-Bertani (LB) medium**: 25 g LB broth powder (Aladdin, Shanghai, China) was added into 1 L deionized H_2_O. After autoclaving for 45 min, the LB medium was stored at room temperature.

**Terrific-Broth (TB) medium**: 23.6 g yeast extract, 11.8 g tryptone, 9.4 g K_2_HPO_4_, 2.2 g KH_2_PO_4_, 4 mL glycerol were added and well mixed in 900 mL deionized H_2_O. The system was further supplemented with deionized H_2_O to 1 L (pH ∼ 7.0). The medium was autoclaved for 45 min and stored at 4°C. 2% (m/v) glucose was supplemented to the medium before use.

**BC medium**: 10 g (NH_4_)_2_HPO_4_, 2 g K_2_SO_4_, 0.3 g NaCl, 0.2 g MgSO_4_·7H_2_O, 4 mg FeSO_4_·7H_2_O, 0.9 mg ZnSO_4_·7H_2_O, 0.4 mg CuSO_4_·5H_2_O, 0.2 mg MnSO_4_·H_2_O, 0.8 mg CaCl_2_·2H_2_O, 0.09 mg Na_2_B_4_O_7_·10H_2_O, 0.6 mg Na_2_SeO_3_·5H_2_O, 0.4 mg (NH_4_)_6_Mo_7_O_24_, 0.9 mg (NH_4_)_2_Ni(SO_4_)_2_·6H_2_O were added and well mixed in 900 mL deionized H_2_O. The system was further supplemented with deionized H_2_O to 1 L (pH ∼ 7.0). The medium was autoclaved for 45 min and stored at 4°C. Glucose was supplemented to the medium (final concentration ∼30 mM) before use.

**SBC medium**: 10 g (NH_4_)_2_HPO_4_, 2 g K_2_SO_4_, 0.3 g NaCl were added and well mixed in 900 mL deionized H_2_O. The system was further supplemented with deionized H_2_O to 1 L (pH ∼ 7.0). The medium was autoclaved for 45 min and stored at 4°C. Glucose was supplemented to the medium (final concentration ∼30 mM) before use.

All medium was supplemented with appropriate antibiotics (50 μg/mL kanamycin, 75 μg/mL ampicillin, 100 μg/mL chloramphenicol) when applicable. 1 mM isopropyl-β-D-thiogalactopyranoside (IPTG), 10 mM L-arabinose (Ara), 200 ng/mL anhydrotetracycline (aTc) were used to induce gene expression when applicable. 2% agar was dissolved in the liquid medium to prepare the corresponding agar plate.

#### Buffer system

**Tetramethyl orthosilicate (TMOS) buffer**: 1.52 mL fresh TMOS was mixed with 8.5 mL deionized H_2_O before addition of 10 μL HCl (2 M). The system was shaken for 30 min to guarantee the dissolution of all the components. The system was further supplemented with deionized H_2_O to 50 mL to a final concentration of 0.2 M.

**Phosphate buffered saline (PBS, 10 mM)**: Ten PBS tablets (Solarbio, Beijing, China) were added into 1 L deionized H_2_O and autoclaved for 45 min. The buffer was stored at 4°C.

**PBST buffer**: 0.05% (v/v) Tween 20 (Sangon Biotech, Shanghai) was added in 200 mL PBS buffer (10 mM) and autoclaved for 45 min. The buffer was stored at 4°C.

### Method details

#### Immunostaining

A single colony was picked and inoculated in 4 mL LB culture medium supplemented with 75 μg/mL ampicillin and 200 ng/mL aTc. The cell culture was then incubated at 37°C for 24 h (shaking speed = 220 rpm). Cells were harvested by centrifugation (2 mL cell culture, 4000 g for 5 min at 4°C). The collected cells were further washed with PBST buffer for three times and resuspended in the same buffer. The primary antibody (Abcam, AB18184, Mouse mAb to 6×His tag) was added into the cells (1:1000 (v/v)). The system was incubated at 4°C for overnight and washed for three times with PBST buffer. The washed system was further supplemented with the secondary antibody (Sangon Biotech, Cy3-conjugated Goat anti-mouse lgG, 1:300 (v/v)), and incubated at 4°C (3 h) followed by 25°C (1 h), respectively. After washing, Hoechst 33342 staining solution (Solarbio, Beijing, China) was supplemented at 1:100 (v/v), and incubated at the room temperature for 15 min. The cells were collected, washed and resuspended in 100 μL PBS. 2 μL resuspended cells were transferred onto the slide and observed by a confocal microscopy (Nikon AX). The excitation and emission were 550/570 nm for Cy3 and 365/480 nm for Hoechst 33342 staining, respectively. For the Hoechst staining, the brightness and the contrast of the image was increased by 40% and 20%, respectively. For the Cy3 staining, the brightness of the image was increased by 40%, while the contrast of the image was decreased by 40%, respectively (Figures 2C and S2).

#### Flow cytometry

1 mL collected cells was re-suspended in PBS with primary antibody (Abcam, AB1206, FITC Anti-6×His tag) (1:300 (v/v)), and incubated in the 4°C overnight. The cells were then collected and then incubated with 1 mL Hoechst 33342 staining solution (Solarbio, Beijing, China) (1:100 (v/v)) at the room temperature for 15 min. The system was washed 3 times with PBST buffer and diluted 500 times in PBS. The stained samples were then analyzed by flow cytometry (Beckman Coulter, CytoFLEX S). Fluorescence was measured for >100,000 events for each sample. We used the CytExpert software for data collection and analysis. FITC-H and PB450-H channels were chosen for measurement, respectively. Data were processed using FlowJo (TreeStar) to obtain the median of fluorescence.

#### Biosilicification

A single colony was picked and inoculated into 25 mL LB medium supplemented with 75 μg/mL ampicillin and 200 ng/mL aTc. The cell culture was then incubated at 37°C for 24 h (shaking speed = 220 rpm). Cells were harvested by centrifugation (25 mL cell culture, 4000 g for 5 min at 4°C). The collected cells were further washed with PBS buffer for three times and resuspended in 5 mL PBS. TMOS buffer was added into the cell culture at 1:5 (v/v). The system was stirred at 500 g for 30 min before further analysis.

#### SEM sample preparation and characterization

After silicification, the cell culture was first diluted by 10 times with deionized water. 10 μL diluted culture was incubated with 400 μL glutaraldehyde fixing solution at 4°C for 2 h. After centrifugation (4000 g, 5 min), the samples were dehydrated by treating with a series of ethanol solutions with a concentration of 50%, 70%, 90% and 100%, each for 5 min, respectively. The system was sequentially resuspended in a mixture of tert-butanol and ethanol (1:1, v/v) and 100% tert-butyl butanol (2 times). Cells were finally collected by centrifugation (4000 g, 5 min). 10 μL cell culture was dripped onto the conductive tape and air-dried for overnight before the SEM examination.

#### Affinity of R5 peptide to silica microsphere

A single colony (BL21(DE3)(p15A-6×His-R5)) was picked and inoculated into 100 mL LB medium supplemented with 50 μg/mL kanamycin. The system was cultured at 37°C (shaking speed = 220 rpm). IPTG (final concentration = 1 mM) was added into the system when OD_600_ attained 0.6–0.8, and the system was then cultured for overnight (37°C, shaking speed = 220 rpm). Cells were harvested by centrifugation (100 mL cell culture, 12000 g for 5 min at 4°C), washed with PBS buffer for 3 times, and re-suspended with 50 mL PBS. Cells were lysed by a sonicater before purification. cOmplete™ His-Tag purification resins (Sigma-Aldrich) were used for purification. After elusion, the buffer was exchanged by ultrafiltration for 3 times to remove the imidazole. 20 μL purified protein solution (∼2.4 mg/mL) and 20 μL silica microsphere solution (Meryer (Shanghai, China), diameter ∼5 μm, 2.5% (w/v)) were incubated at 4°C for overnight. As control, 20 μL silica microspheres were mixed with 20 μL ddH_2_O and incubated at 4°C for overnight. 10 μL of each system was dripped onto the conductive tape respectively, dried at room temperature and then observed under the SEM (Phenom XL).

#### Evaluation on affinity of R5 to silica microsphere

To test the affinity of R5 peptide to the silica microspheres, two plasmids (R5-RFP and RFP) were constructed and transformed into *E*. *coli* BL21 (DE3), respectively. A single colony was picked up and inoculated into 100 mL LB medium supplemented with 50 μg/mL kanamycin. The system was cultured at 37°C (shaking speed = 220 rpm). IPTG (final concentration = 1 mM) was added into the system when OD_600_ attained 0.6∼0.8, and the system was then cultured for overnight at 30°C (shaking speed = 220 rpm). Cells were harvested by centrifugation (100 mL cell culture, 12000 g for 5 min at 4°C), washed with PBS buffer for three times, and re-suspended with 50 mL PBS. Cells were lysed by sonicater before purification. cOmplete™ His-Tag purification resins (Sigma-Aldrich) were used for purification. After elusion, the buffer was exchanged by ultrafiltration for 3 times to remove the imidazole. 100 μL silica microsphere solution (2.5% (w/v)) was incubated with 500 μL purified R5-RFP or 500 μL RFP solutions (∼2.4 mg/mL), respectively at 4°C for 30 min. The silica microspheres were collected by centrifugation at 500*g* for 5 min, and washed by PBS for three times. The system was resuspended in 100 μL PBS. 2 μL solution was dripped onto the glass slide and observed by a confocal microscopy (Nikon AX) at 520/610 nm (excitation/emission).

#### Size quantification of R5-silica aggregation

To analyze the affinity of R5 to silica microsphere quantitatively, SiO_2_ microsphere samples (Meryer (Shanghai, China), grain size ∼200 nm, 2.5% w/v) was diluted with PBS by 200 times to a final volume of 1 mL. Then, SiO_2_ microsphere samples were further diluted with PBS containing purified R5 protein solution (final R5 concentration: ∼1.2 mg/mL, R5+) or just PBS (R5-). Both samples were incubated in the room temperature for 1 h and the size of the resultant system was analyzed by dynamic laser light scattering (Malvern Zetasizer Pro, software version: Zetasizer 2.3.1.4). The temperature is set as 25°C and the thermal equilibrium time is 120 s during measurement, all the samples were tested 3 times.

#### Evaluation on affinity of R5 to standard sand

0.1 g standard sand was washed by PBS for three times, and then grinded in a mortar. The grinded system was washed again with PBS for three times and resuspended in 200 μL PBS. 20 μL standard sand solution was incubated with 100 μL purified R5-RFP or RFP solutions at 4°C for 30 min. The standard sand was then collected by centrifugation at 500*g* for 5 min. The system was washed with PBS by three times and re-suspended with 100 μL PBS. 2 μL sample was dripped onto the glass slide and observed by a confocal microscopy (Nikon AX) at 520/610 nm (excitation/emission).

#### Cell settlement dynamics tracking experiment

A single colony was picked and inoculated into 25 mL LB medium supplemented with 75 μg/mL ampicillin and 200 ng/mL aTc. The cell culture was then incubated at 37°C for 24 h (shaking speed = 220 rpm). Cells were harvested by centrifugation (25 mL cell culture, 4000 g for 5 min at 4°C). The collected cells were further washed with PBS buffer for three times and resuspended in 5 mL PBS. 1 mL TMOS buffer was added into the system to initiate silicification. OD_600_ value of the supernatant in the reaction system was measured every 5 min to track the sedimentation dynamics.

#### Living building materials assembly

The Chinese ISO standard sand was purchased from Xiamen Aisio Standard Sand Co., Ltd. The standard sand was washed by PBS before usage. The shape of the mold was designed and drawn by SolidWorks and then sent for fabrication. The mold with a bottom area of 1 cm × 1 cm was used to prepare cube samples for compressive test, while the mold with a bottom area of 1 cm × 6 cm was used to prepare samples for three-point bending test. Compressed or non-compressed samples were prepared in steel mold or silicone mold, respectively.

The R5 displayed cells were cultured, induced and harvested as described above. The cells were centrifuged and washed three times (with PBS, 10 mM) at 4000 g, and finally collected at 8000 g for the subsequent construction of bio-bricks. The collected cells (3.6 g) were thoroughly mixed with 20 g, 40 g, 80 g or 120 g standard sand in a culture dish. After 1 h, the mixture was added to a steel mold lined with a stainless steel mesh (the diameter of mesh and wire is ∼0.038 mm and ∼0.030 mm, respectively). The stainless steel mesh on the upper and lower surfaces of the mixture facilitated the subsequent mold release. The mold was then placed on the powder compaction machine (DY-20, Tianjin Keqi High Tech Co., Ltd), which compressed the mold with a certain amount of pressure (1 ton, 2 tons or 4 tons for preparing cube samples, and 6 tons or 12 tons for preparing samples of three-point bending tests). The samples were then released from the mold and placed at room temperature for moisture evaporation. Daily monitoring was conducted to track the changes in cubic mass.

To generate the non-compressed samples, the mixture of cells and sand were shaped in the silicone mold. The mixture of sand and cells expressing R5 and various fluorescent proteins was used to create bricks with customizable colors in silicone mold (the mixture ratio of cells to sand is 3.6:20 (w/w)). ∼ 21.6 g R5-displayed cells (expressing mScarlet or mOrange protein, 1:1 cell ratio) were mixed with 600 g sand to fabricate a 10 cm × 10 cm × 3 cm bio-brick. The combined brick was prepared by applying R5/mScarlet-expressing cells (∼2 g) as a binder on the contact surfaces of three rectangular bio-bricks (6 cm × 1 cm × 1 cm, the mixture ratio of cells to sand is 3.6:20 (w/w)) after air-drying. The blank control samples used PBS (10 mM) instead of cells to mix with sand.

All samples mentioned above were air-dried in the laboratory environment, except for the large bio-blocks, which were dried in a 37°C oven. Samples prepared using silicone mold were taken out after air-drying, while samples prepared using the powder compaction machine were taken out from steel mold immediately after pressing.

#### Compressive test and three-point flexural test

The samples were tested for mechanical properties at the third day after preparation. The Bluehill Universal testing machine (68SC-2, 2kN) was used for the compressive test. The displacement-controlled loading rate was set at 0.5 mm/min. The Shimadazu testing machine (AG-X plus, 500N) was used for the three-point flexural test. The displacement-controlled loading rate was set at 0.2 mm/min. To ensure the flatness of the load-bearing surfaces of the test specimen, the front and back surfaces of the fabricated sample were placed in direct contact with the testing fixture.

#### Assembly of structured aggregation

A single colony (BL21(DE3)(R5/RFP) or BL21(DE3)(R5/GFP)) was picked and inoculated into 4 mL LB medium supplemented with 75 μg/mL ampicillin and 50 μg/mL kanamycin. The cell culture was then incubated at 37°C for overnight (shaking speed = 220 rpm). Then, 500 μL overnight culture of each was inoculated into 50 mL fresh LB medium with 200 ng/mL aTc and incubated at 37°C for 24 h (shaking speed = 220 rpm). IPTG (final concentration = 1 mM) was added into the system when OD_600_ grew to 0.6 ∼ 0.8. Cells were harvested by centrifugation (50 mL cell culture, 4000 g for 5 min at 4°C), washed with PBS buffer for 3 times and re-suspended with 10 mL PBS.

To assemble the structured aggregation, 5 mL collected cells (BL21(DE3)(R5/GFP), cells in the core region) were first mixed with 1 mL TMOS buffer and stirred at 500 g for 30 min. After the sedimentation, 5 mL collected cells (BL21(DE3)(R5/RFP), cells in the shell region) and 1 mL TMOS buffer was added in turn, and stirred at 500 g for another 30 min. After the cell settlement, the aggregation was washed by PBS for 3 times. For non-structured group, 5 mL collected cells (BL21(DE3)(R5/GFP)) and 5 mL collected cells (BL21(DE3)(R5/RFP)) were first well-mixed. 2 mL TMOS buffer were further added and the system was stirred at 500 g for 30 min. After sedimentation, the aggregation was washed by PBS for 3 times.

To characterize the structure, 50 μL cell aggregation was transferred onto the confocal culture dish and imaged by a confocal microscopy (Nikon AX) at 480/520 nm (GFP excitation/emission), and 580/610 nm (RFP, excitation/emission), respectively. The brightness was increased by 20% for all the images in Figures 5B and S13.

#### 1-Butanol biomanufacturing

A single colony BL21(DE3)(R5/pb12) was picked and inoculated into 4 mL LB medium supplemented with 100 μg/mL chloramphenicol and 75 μg/mL ampicillin. The cell culture was then incubated at 37°C for overnight (shaking speed = 220 rpm). Then, 1 mL overnight culture was inoculated into 100 mL fresh LB medium. Arabinose and aTc was supplemented at a final concentration of 10 mM and 200 ng/mL, respectively, when OD_600_ reached ∼0.6. The system was further incubated at 37°C for 24 h (shaking speed = 220 rpm). 100 mL cells were harvested by centrifugation (4000 g for 10 min, at 4°C), washed with PBS buffer for 3 times, and re-suspended with 20 mL PBS.

To generate the structured aggregation (cells carrying the pathway in the core region), 20 mL BL21(DE3)(R5/pb12) were first mixed with 4 mL TMOS buffer and stirred at 500 g for 30 min. After the sedimentation, 20 mL BL21(DE3)(R5) and 4 mL TMOS buffer were added in turn and stirred at 500 g for another 30 min. After the cell settling, the aggregation was washed by PBS for 3 times. The aggregates were then transferred to 50 mL TB medium with 0.04 g/L glucose and 10 mM arabinose in a 100 mL glass reaction flask. The reaction was conducted either anaerobically (blowing nitrogen for 30 min to deplete the oxygen before seal) or aerobically (cap-open for 30 min) at 37°C for 72 h. The product was further analyzed by gas chromatography.

#### Product isolation and gas chromatography (GC) measurement

1-butanol was extracted by an equivalent volume of ethyl acetate and incubated in tube with drastic shaking. The organic phase was then harvested by centrifugation (12000 g for 2 min) for further usage. Products were analyzed using GC (Agilent GC 8890, Agilent Technologies) equipped with a DB-WAX column (Agilent 125–7032, 30 m × 530 μm × 1 μm). The carrier gas was nitrogen. 1-butanol was separated in GC via temperature programming as follows: held at initial temperature of 40°C for 4 min, increased the temperature to 70°C at a rate of 5 °C/min. The total run time was 10 min. To quantify the yield in different reaction group, the standard 1-butanol in different concentrations (31.25, 62.5, 125, 250 and 500 mg/L) were analyzed by GC. We then generated the calibration curve by plotting the accumulated peak area against the known concentration. The yield of different reaction groups was then calculated back based on the calibration curve. The sample injection volume was 1 μL.

#### Hydrogen biomanufacturing

A single colony of BL21(DE3)(R5/hyaABCDEF) was picked and inoculated into 4 mL LB medium supplemented with 50 μg/mL kanamycin and 75 μg/mL ampicillin. The cell culture was then incubated at 37°C for overnight (shaking speed = 220 rpm). Then, 1 mL overnight culture was inoculated into 50 mL fresh LB medium. IPTG and aTc were supplemented at a final concentration of 1 mM and 200 ng/mL, respectively, when OD_600_ reached ∼0.6. Then system was further incubated at 37°C for 12 h (shaking speed = 220 rpm). 50 mL cells were harvested by centrifugation (4000 g for 10 min at 4°C), washed with PBS buffer for 3 times, and re-suspended with 50 mL BC medium containing 30 mM glucose. The cells were incubated at 30°C for 12 h under anaerobic condition (shaking speed = 220 rpm). 50 mL cells were harvested by centrifugation (4000 g for 10 min at 4°C) again, washed with PBS buffer for 3 times and re-suspended with 10 mL PBS buffer.

To generate the structured aggregation (cells carrying the pathway in the core region), 10 mL BL21(DE3)(R5/hyaABCDEF) were first mixed with 2 mL TMOS buffer and stirred at 500 g for 30 min. After the sedimentation, 10 mL BL21(DE3)(R5) and 2 mL TMOS buffer was added in turn, and stirred at 500 g for another 30 min. After the cell settlement, the aggregation was washed by PBS for 3 times. The cells were then transferred to 20 mL SBC medium with 30 mM glucose in a 50 mL top-window quartz reaction cell. The reaction was conducted either anaerobically (blowing nitrogen for 30 min to deplete the oxygen before seal) or aerobically (cap-open for 30 min) at 30°C for 24 h. The product was further analyzed by gas chromatography.

#### Gas chromatography (GC) measurement of hydrogen

Products were analyzed using gas chromatography (GC 9790II, FuLi Instruments) equipped with a 5A molecular sieve column (SJ20232145P, 60–80 mesh, Dalian Sanjie Scientific Development Co.Ltd). The carrier gas was argon. Hydrogen was separated in GC via temperature programming as follows: the temperature of column oven and the temperature of TCD was held at 80°C and 150°C. The total run time was 4 min. To quantify the yield in different reaction group, the standard of hydrogen was diluted to 5 mM and analyzed by GC. The hydrogen yield of different reaction groups was calculated back based on the accumulated peak area ratio of the sample and the standard. The sample injection volume was 100 μL.

#### Model equations and justifications

We developed a kinetic model to examine how silicified cells aggregate and its dependence on the initial cell number. The model comprises a set of ordinary differential equations that describe the temporal dynamics of aggregated species A_j_ (j = 1,2,…N). Here, N is the maximum number of cells in the aggregates. We first take into account the silicification step, where the R5 displayed cells (C) attach with Si(OCH_3_)_4_ (denoted as S) and switch into aggregation prone state (A_1_). The aggregation is initiated by the dimerization of two A_1_ monomers and proceeds with forming trimer, tetramer and j-mers. The reaction schemes are:

$$C+S\begin{array}{c} k_{p1}\\ ⇄\\ k_{n1} \end{array}A_{1}$$

$$A_{1}+A_{1}\begin{array}{c} k_{p\left(1,1\right)}\\ ⇄\\ k_{n\left(1,1\right)} \end{array}A_{2}$$

$$A_{i}+A_{j}\begin{array}{c} k_{p\left(i,j\right)}\\ ⇄\\ k_{n\left(i,j\right)} \end{array}A_{i+j}$$

where A_j_ represents the abundance of the j-mer. $k_{p\left(i,j\right)}$ stands for the rate at which A_i_ and A_j_ aggregate, and $k_{n\left(i,j\right)}$ is the rate of the reverse reaction. The temporal dynamics of the system can then be described by the following ODEs:

$$\frac{dC}{dt}=-k_{p1}C\cdot S+k_{n1}A_{1}\text{,}$$

$$\frac{dA_{1}}{dt}=k_{p1}C\cdot S-k_{n1}A_{1}-2k_{p\left(1,1\right)}A_{1}^{2}+2k_{n\left(1,1\right)}A_{2}+\sum_{j=3}^{N}\left(-k_{p\left(j-1,1\right)}A_{j-1}A_{1}+k_{n\left(j-1,1\right)}A_{j}\right)\text{,}$$

$$\frac{dA_{j}}{dt}=\sum_{i=1}^{j/2}\left(k_{p\left(i,j-i\right)}A_{i}A_{j-i}-k_{n\left(i,j-i\right)}A_{j}\right)-\sum_{m=1}^{N-j}\left(k_{p\left(m,j\right)}A_{m}A_{j}-k_{n\left(m,j\right)}A_{m+j}\right)-k_{p\left(j,j\right)}A_{j}^{2}\cdot \phi \left(j\right)+k_{n\left(j,j\right)}A_{2j}\cdot \phi \left(j\right),2\leq j\leq N-1\text{,}$$

$$\frac{dA_{N}}{dt}=\sum_{i=1}^{N/2}\left(k_{p\left(i,N-i\right)}A_{i}A_{N-i}-k_{n\left(i,N-i\right)}A_{N}\right)\text{.}$$

Here, ϕ(j) is a logic function and defined as:

$$\phi \left(j\right)=\left\{ \begin{array}{c} 1,j\leq N/2\\ 0,j\geq N/2 \end{array}\right.$$

When the values of the kinetic parameters are given (Table S1), this model allows us to numerically simulate the dynamics of cell aggregation.

### Quantification and statistical analysis

Statistical analysis was performed using Excel (2013). Details of specific analyses and statistic tests are described in applicable figure legends.

## Supplemental information

Document S1. Figures S1–S15, and Tables S1 and S3

Table S2. Protein sequences

Data S1. Source image data for main and supplementary figures
