# Supplementary material for: Programmable cell aggregation by a synthetic biosilicification approach
Source: iScience. 2025 May 26;28(6):112519. doi: 10.1016/j.isci.2025.112519 (PMC12182305; doi:10.1016/j.isci.2025.112519)
Supplement: Document S1. Figures S1–S15, and Tables S1 and S3 [file mmc1.pdf]

iScience, Volume *28*

Supplemental information

Programmable cell aggregation

by a synthetic biosilicification approach

Qing Wang, Jing Sun, Lei Zang, Huaxiong Yao, Lin Wang, Runtao Zhu, Jie Li, Simin Zeng, Hongting Tang, Teng Wang, Ji Liu, Bo Wang, Bo Li, Zhiyuan Liu, and Zhuojun Dai

Supplementary Materials for this manuscript include the following:

Supplementary Figures 1-15

Supplementary Table 1 and 3

Supplemental Figures

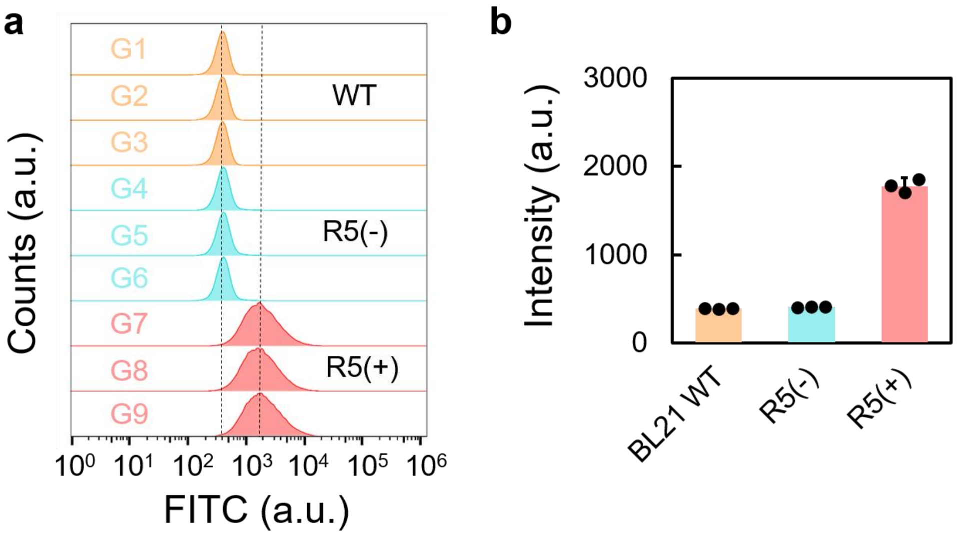

Supplementary Figure 1. Cell engineering for R5 peptide display.

a. **Flow cytometry results suggested the successful display of R5 peptide on induced cells.** WT, R5 (-) and R5 (+) represent wide type cells (without the circuit), un-induced and induced cells (both carrying the circuit), with 100,000 cells counted per sample. This figure includes additional biological replicates to **Figure 2b**. Experiments were repeated independently more than three times with similar results.

b. **Quantification of median fluorescence intensity verified the successful display of R5 peptide on induced cells.** Column = mean value (n = 3). Data are represented as mean ± standard deviation.

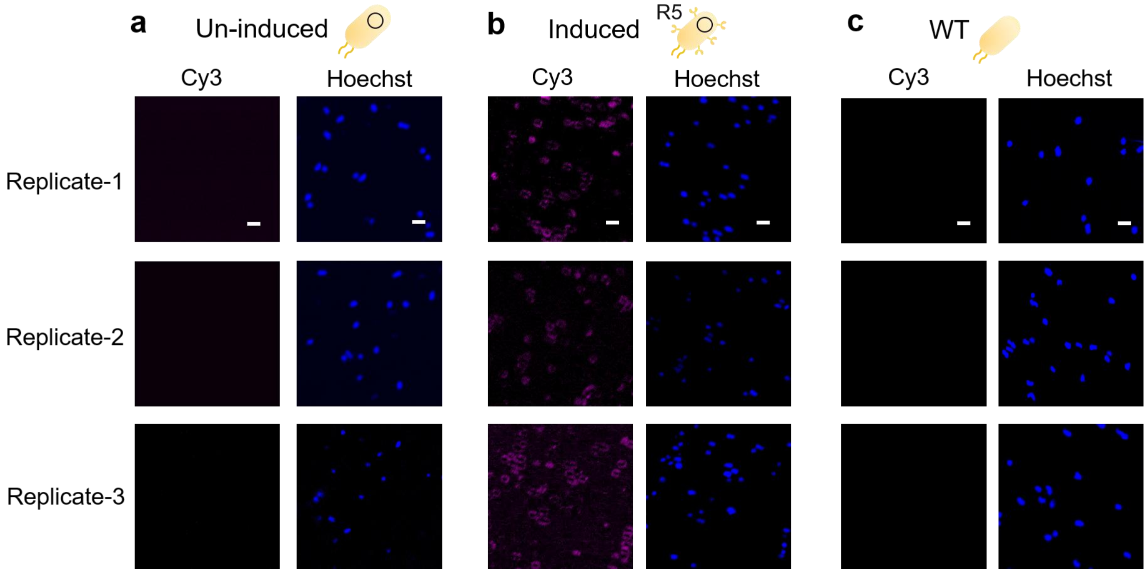

Supplementary Figure 2. Cell engineering for R5 peptide display regulated by an inducible gene circuit. Immunostaining analysis of un-induced (a), induced (b) and wild-type (c) bacteria suggested the presence of R5 on the surface of induced cells. Fluorescence was captured in both the Hoechst and Cy3 channels for induced cells, representing Hoechst 33342 staining (a universal DNA dye) and immunostaining for the His-tagged R5, respectively. This figure presents additional biological replicates to **Figure 2c**. Scale bar = 2 μm. Experiments were repeated independently more than three times with similar results.

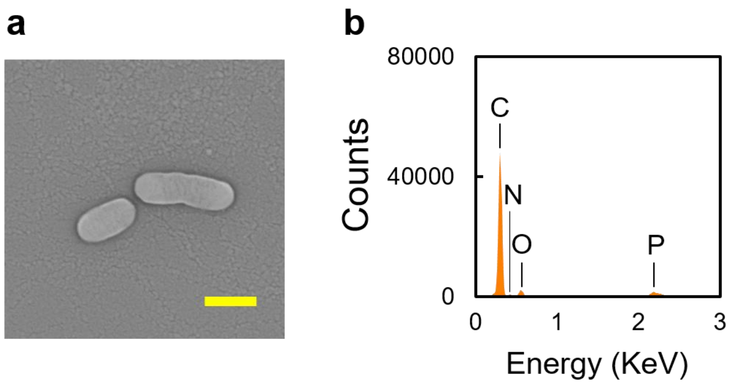

Supplementary Figure 3. No silica deposition on the wild-type cells.

a. **No biosilica deposition was observed in SEM.** Image shows surface morphology of wild-type cells after incubation with TMOS buffer. Scale bar = 1 μm.

b. **Energy dispersive spectroscopy (EDS) analysis confirmed no silicon presence on the cell surface of wild-type cells after incubation with TMOS buffer.**

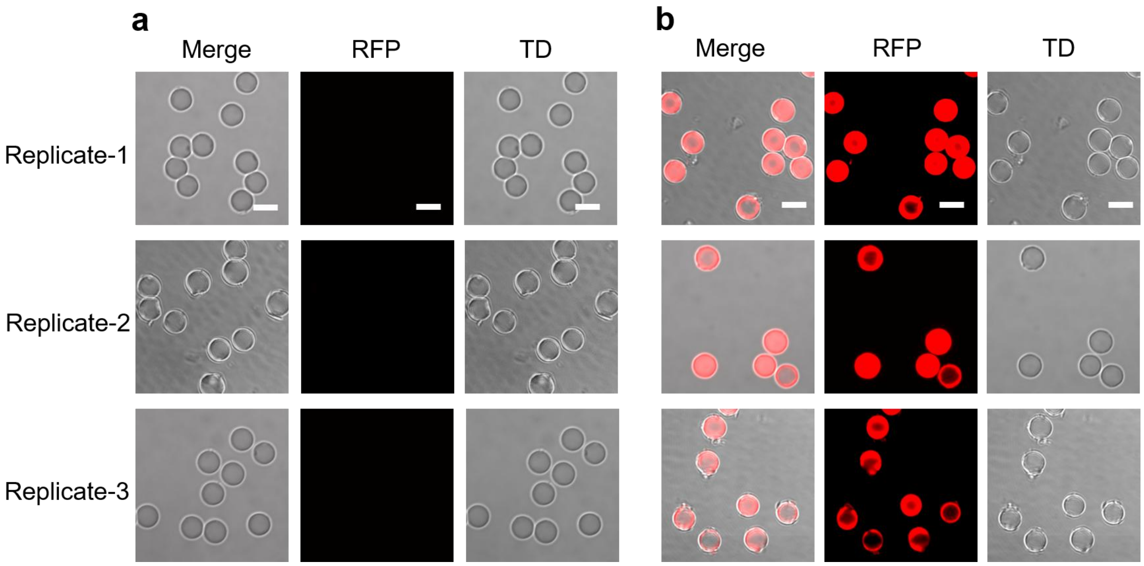

Supplementary Figure 4. Microscopy confirmation of R5-fused RFP conjugation to silica microspheres.

a. **No fluorescence was observed when RFP alone was incubated with silica microspheres.** Columns represent merged, RFP and transmitted light channels, and rows indicate biological replicates. This figure presents additional biological replicates to **Figure 3b**. Scale bar = 5 μm.

b. **Fluorescence was detected when R5-fused RFP was incubated with silica microspheres.** Columns represent merged, RFP and transmitted light channels, and rows indicate biological replicates. This figure includes additional biological replicates to **Figure 3b**. Scale bar = 5 μm.

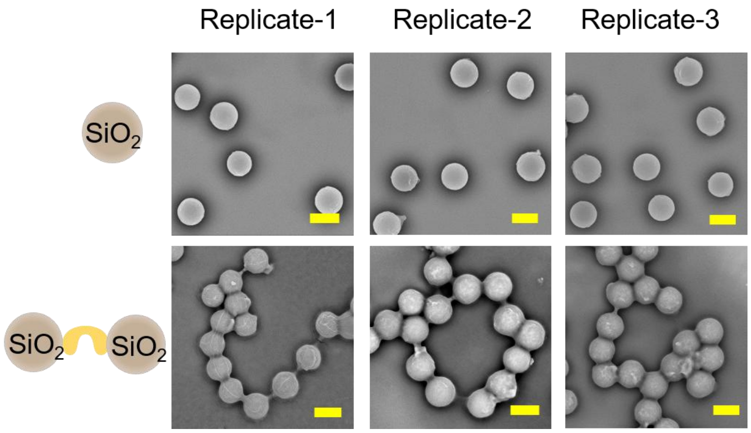

Supplementary Figure 5. Clustering of silica microspheres promoted by the purified R5 peptide. SEM analysis suggested that purified R5 drove the aggregation of silica microspheres. The top row shows silica microspheres incubated with deionized H_2O, while the bottom row shows microspheres incubated with purified R5. Columns represent biological replicates. This figure presents additional biological replicates to **Figure 3c**. Scale bar = 5 μm.

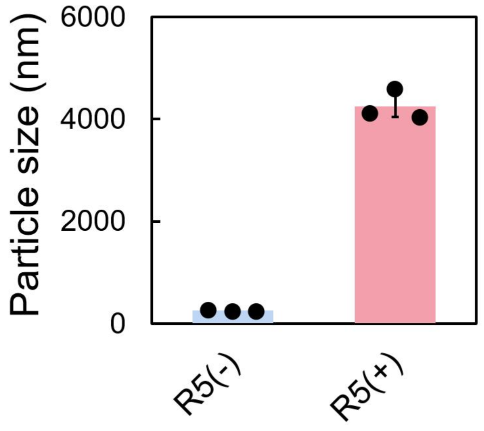

Supplementary Figure 6. Clustering of silica microspheres promoted by the purified R5 peptide quantified by dynamic laser light scattering (DLS). DLS analysis suggested that purified R5 drove the aggregation of silica microspheres. The average of the particle size increased from 254 nm to 4254 nm, with the addition of purified R5. Column = mean value (n = 3). Data are represented as mean ± standard deviation.

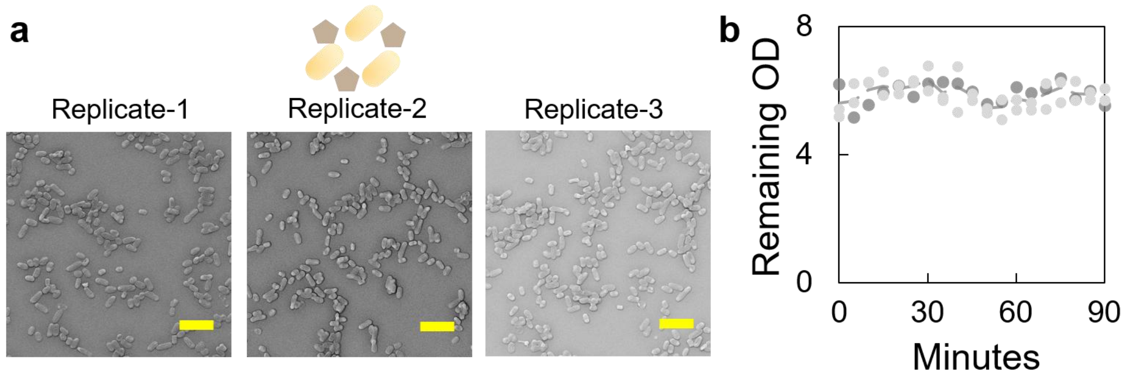

Supplementary Figure 7. No aggregation observed in wild-type cells incubated with TMOS buffer.

a. SEM images show wild-type cells remaining dispersed after incubation with TMOS buffer. Scale bar = 4 μm.

b. Absence of cell settlement in wild-type cells. No cell settlement was detected in wild-type cells incubated with TMOS buffer, as indicated by OD_{600} measurements in the supernatant taken every 5 minutes. Dashed line = mean value (n = 3).

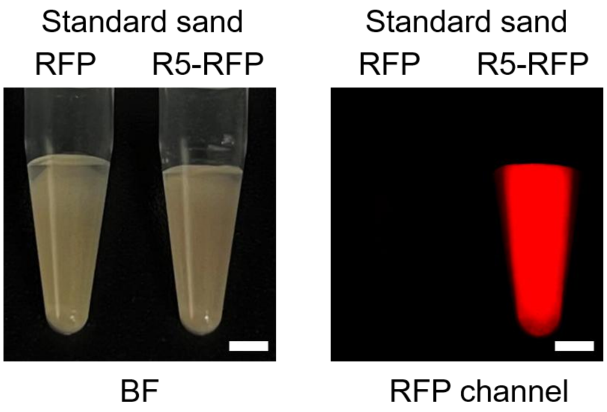

Supplementary Figure 8. Affinity of R5 fused proteins to standard sand. Incubating RFP with sand led to no detectable fluorescence under observation (left tube). In contrast, the binding of R5-RFP to sand produced observable fluorescence (right tube). Scale bar = 2 mm.

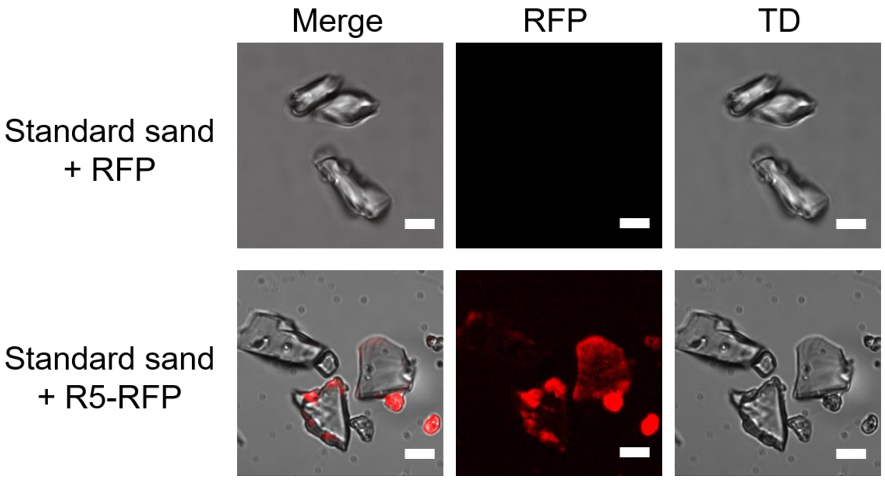

Supplementary Figure 9. Microscopy confirmation of R5-RFP conjugation with standard sand. The top row shows standard sand incubated with RFP, while the bottom row shows standard sand incubated with R5-fused RFP. Columns represent merged, RFP and transmitted light channels, respectively. Scale bar = 5 μm.

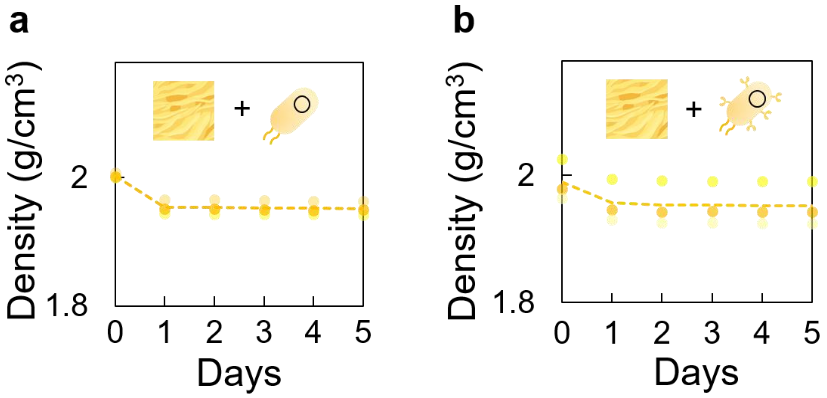

Supplementary Figure 10. Moisture evaporation in bio-bricks. Excess moisture in the bio-brick cubes (1 cm × 1 cm × 1 cm) largely evaporated within two days after preparation. Samples were molded under a pressure of 4 tons. Panels **a** and **b** show cubes made with un-induced and induced cells, respectively, with a cell-to-sand ratio of 3.6:20 (w/w). Dashed line = mean value (n = 3).

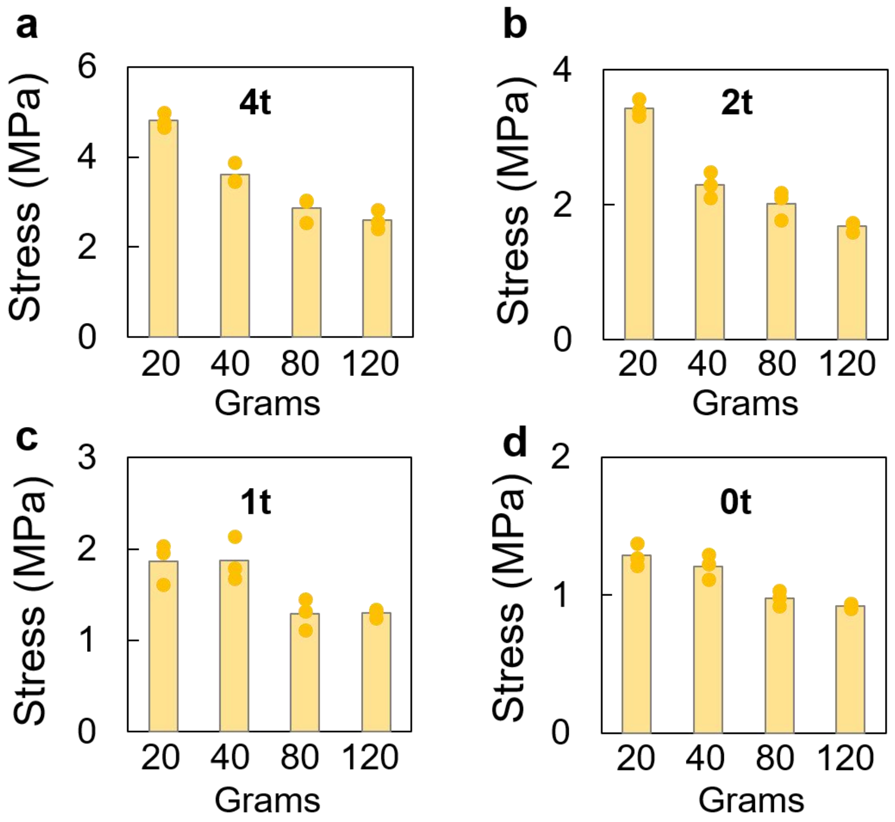

Supplementary Figure 11. Compressive strength assessment of bio-bricks at various sand-to-binder ratios. Compressive strength of bio-bricks was evaluated by adjusting the sand-to-binder ratio while keeping the binder weight (induced cells) constant at 3.6 grams. Strength was tested under varying compression stresses during preparation (from 4 tons to 0 tons, panels **a-d**), with higher binder-to-sand ratios leading to increased strength. Column = mean value (n = 3). Experiments were repeated independently more than three times with similar results.

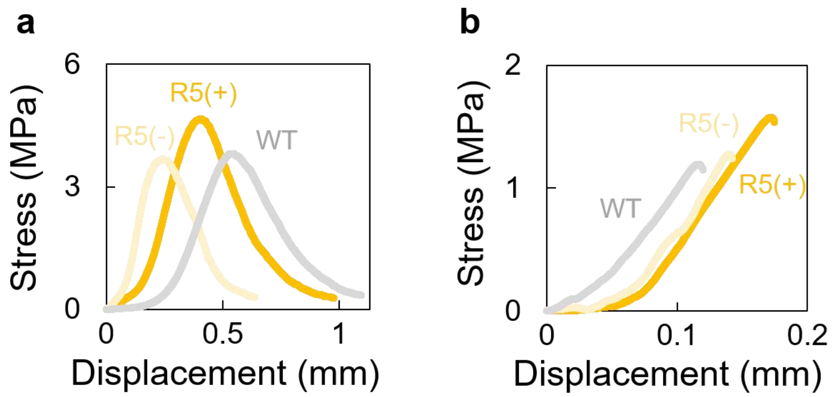

Supplementary Figure 12. Testing curves for compression (a) and three-point bending (b) experiments. Compression samples (**a**) were molded into cubes under 4 tons of pressure. Three-point bending samples (**b**) were molded into cuboids under 12 tons of pressure. The cell-to-sand ratio in both cases was 3.6:20 (w/w).

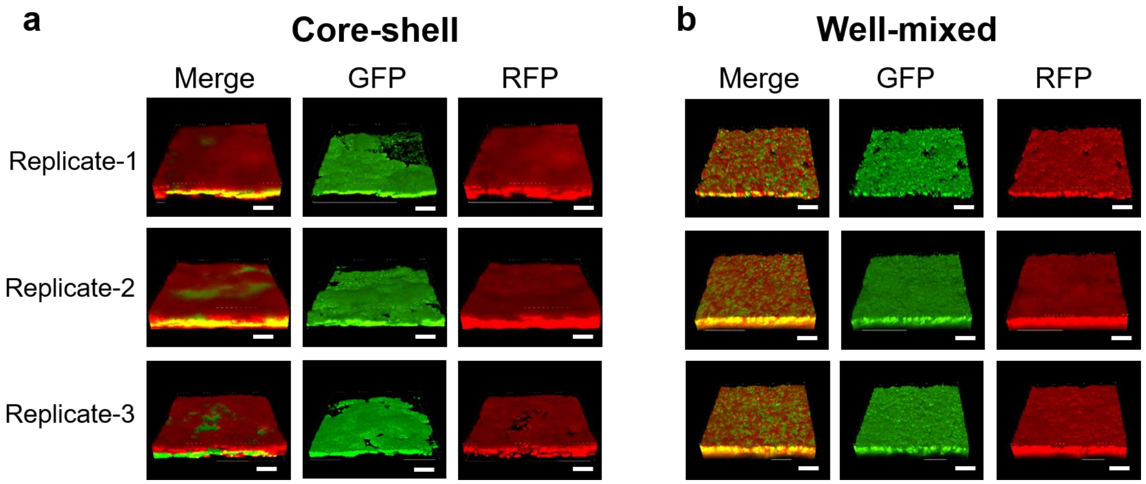

Supplementary Figure 13. Generating the structured aggregation by sequential layering the silicified cells.

a. **Generating the structured aggregation by sequential layering the cells.** Cells expressing R5/GFP (cells in the core) were first sedimented through biosilicification, followed by the addition and settlement of R5/RFP cells (cells in the shell) to create the structured aggregation.
b. **Simultaneous settling of both cell types resulted in uniformly mixed fluorescence**. Scale bar = 10 μm.

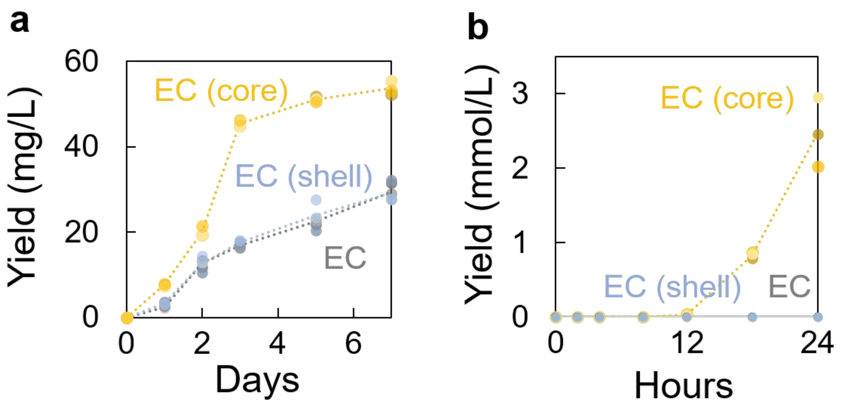

Supplementary Figure 14. Time-dependent production yields of 1-butanol and hydrogen under aerobic condition.

a. 1-Butanol production. Engineered cells in the core layer (EC (core), cells carrying the pathway in the core layer of the aggregate) produced higher 1-butanol yields over time compared to cells in the shell layer (EC (shell), cells carrying the pathway in the shell layer) and unstructured engineered cells (EC, cells carrying the pathway). Dotted line= mean value (n=3).

b. Hydrogen production. Similarly, engineered cells in the core layer (EC (core)) generated more hydrogen over time than cells in the shell layer (EC (shell)) and unstructured engineered cells (EC). Dotted line= mean value (n=3).

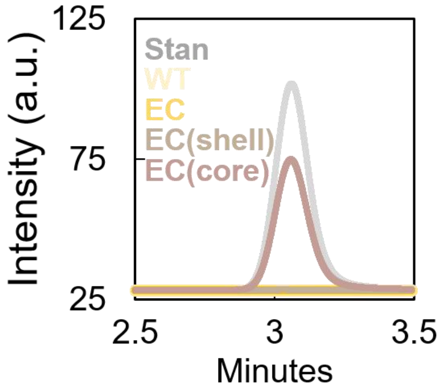

Supplementary Figure 15. Detection of hydrogen by GC with engineered cells in the core layer of the aggregate under aerobic condition. Different curves present a standard (Stan), wild-type cells (WT, no pathway), engineered cells (EC, cells carrying the pathway), engineered cells at shell layer (EC at shell, cells carrying the pathway in the shell layer of the aggregate) and engineered cells at core layer (EC at core, cells carrying the pathway in the core layer of the aggregate).

Supplemental Tables

Supplemental Table 1: Definitions and the values of parameters used in the model

Parameter	Definition	Value
S	The concentration of $Si(OCH_3)_4$ (constant)	0.2
k_{p1}	Rate constant for silification forward reaction (1/s)	0.1
k_{n1}	Rate constant for silification reverse reaction (1/s)	0.08
$k_{p(i,j)}$	Rate constant for aggregation forward reaction (1/nmol•s)	$10^{1.5}$
$k_{n(i,j)}$	Rate constant for aggregation reverse reaction (1/s)	10^{-2}

Supplementary Table 3. Oligos for plasmid assembly

**Oligos**	**Sequence**
p15A-F	taaggtctcaccccctagca
p15A-R	tttttacctccttggtctct
RFP-F	ccaaggaggtaaaaaatggcttcctccgaagacgt
RFP-R	gggggtgagaccttagtggtggtggtggtg
mOrgane-F	ccaaggaggtaaaaaatggtgagcaagggcgagga
mOrgane-R	gttatgctagggggtgagaccctacttgtacagctcgtcca
RFP-R5-F	actccaccggtgctggtggtggtggttccatgtcc
RFP-R5-R	agcaccggtggagtgacga
R5-F	actccaccggtgctggtggtggtggttccatgtcc
R5-R	gggggtgagaccttagtggtggtggtggtgta
mScarlet-F	gaggtaaaaaatggtgagcaagggc
mScarlet-R	tgagaccttacttgtacagctcgtc
Nb-F	ccaaggaggtaaaaaatgcaggtgcagctacaggagtctg
Nb-R	ggaaccaccaccacctgaggagacggtgacctggg
GFP-F	gaccaaggaggtaaaaaatgcgtaaaggcgaagag
GFP-R	tgctagggggtgagaccttatttgtacagttcatccatac
Ec-atoB-F	agatcttttaagaaggagatatacatatgaaaaattgtgtcatcgtcagtgcgg
Ec-atoB-R	actttcattttgctttcctccttcattaattcaaccgttcaatcaccatcgc
Ca-adhE2-F	tgaaggaggaaagcaaaatgaaagttacaaatcaaaaagaactaaaacaa
Ca-adhE2-R	ttagttccatgatgttttcctcctactagttaaaatgattttatatagatatcc
Crt-F	ctagtaggaggaaaacatcatggaactaaacaatgtcatccttgaaaa
Crt-R	ttcatggttatttcctcctagatcctcactatctatttttgaagccttcaat
hbd-F	tgaggatctaggaggaaataaccatgaaaaaggtatgtgttataggtg
hbd-R	aatcatatgatctccctcctagatccttattttgaataatcgtagaaacctt
ter-F	ggatctaggagggagatcatatgattgtaaaaccaatggttaggaaca
ter-R	atgcctggagatccttactcgagtttggatccttaaatcctgtcgaacctttctacctcg
T7-vector-F	agctgagttggctgct
T7-vector-R	gctgctgcccatggtata
hy-F	gatataccatgggcagcagcatgaataacgaggaaacattttaccagg
hy-R	tggcagcagccaactcagctttttacgtcggtgca
